# The Memorial to Florence Nightingale

**Published:** 1911-11-11

**Authors:** 

**Affiliations:** Wilton House, Salisbury


### The Editor's Letter Box.

### THE MEMORIAL TO FLORENCE NIGHTINGALE.

The Earl of Pembroke writes from Wilton House, Salisbury: In the early summer you were good enough to allow me to appeal in your columns for subscriptions to a memorial to Miss Florence Nightingale. A meeting was held at the Mansion House, and it was settled that the memorial should take the form of a statue, and any balance be devoted to an annuity fund for nurses who are past work or disabled through illness. But, just at that time, everyone was absorbed with the Coronation and the manifold interests connected with it, so it was decided to let the appeal rest in abeyance until the autumn.

I am happy to announce that the very best—because the most appropriate—site in all London has been granted by the Office of Works for the statue immediately opposite the Guards' Crimean Memorial. The statue of "The Lady with the Lamp" will stand between the Athenæum and Senior United Service Club. A statue will cost about £6,000, and I hope we may have, over and above the cost of the statue, a fund to help the nurses. We have raised so far only £2,800—of which £900 comes from soldiers and £500 has been given by nurses. When the general public realise that this movement has been set on foot I am confident that I shall not appeal in vain. There must be so many men and women who would gladly share in helping to keep alive through future generations the memory of Florence Nightingale's noble personality. It is unthinkable that the memory of Florence Nightingale's name and work should not be perpetuated to all time, and there is no better way of doing this than to have a statue of her in a prominent position in the capital of the country for which she did so much. It is not a mere form of words, or an exaggeration, to say that we owe all our sick nursing and the high standard to which it has now reached to this one woman's example and devotion.

Subscriptions, however small, may be sent to the Honorary Secretary of the Nightingale Memorial Fund, St. Thomas's Hospital, S.E., or direct to me.

